# Tetra­kis(1,3,4,6,7,8-hexa­hydro-2*H*-pyrimido[1,2-*a*]pyrimidin-9-ido-κ^2^ *N* ^1^,*N* ^9^)niobium(V) hexa­fluorido­phosphate

**DOI:** 10.1107/S1600536808026627

**Published:** 2008-08-23

**Authors:** F. Albert Cotton, Carlos A. Murillo, Pavel V. Poplaukhin, Nattamai Bhuvanesh, Edward R. T. Tiekink

**Affiliations:** a Laboratory for Molecular Structure and Bonding, Department of Chemistry, Texas A&M University, PO Box 30012 College Station, Texas 77842-3012, USA; b X-ray Diffraction Laboratory, Department of Chemistry, Texas A & M University, PO Box 30012 College Station, Texas 77842-3012, USA; c Department of Chemistry, The University of Texas at San Antonio, One UTSA Circle, San Antonio, Texas 78249-0698, USA

## Abstract

The title complex, [Nb(C_7_H_12_N_3_)_4_]PF_6_, features chelating hpp anions (hpp is 1,3,4,6,7,8-hexahydro-2*H*-pyrimido[1,2-*a*]pyrim­idine) that define a distorted dodeca­hedral coordination geometry based on an N_8_ donor set. The Nb atom is situated on a site of symmetry 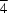, and the PF_6_^−^ anion has crystallographic fourfold symmetry.

## Related literature

For background literature, see: Cotton *et al.* (1998 ▶, 2005 ▶). For related structures, see: Cotton *et al.* (2000 ▶); Coles & Hitchcock (2001 ▶).
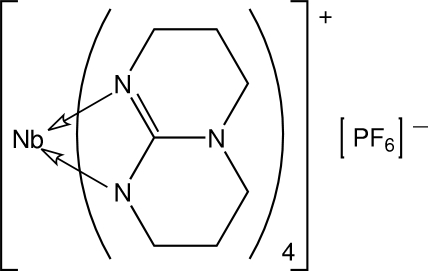


## Experimental

### 

#### Crystal data

[Nb(C_7_H_12_N_3_)_4_]PF_6_
*M* *_r_* = 790.66
Tetragonal, 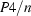

*a* = 13.531 (6) Å
*c* = 9.159 (4) Å
*V* = 1676.9 (13) Å^3^
*Z* = 2
Mo *K*α radiation
μ = 0.48 mm^−1^
*T* = 213 (2) K
0.20 × 0.15 × 0.10 mm

#### Data collection

Bruker SMART 1K CCD area-detector diffractometer
Absorption correction: multi-scan (*SADABS*; Sheldrick, 2004 ▶) *T*_min_ = 0.910, *T*_max_ = 0.953
10356 measured reflections
1655 independent reflections
1381 reflections with *I* > 2σ(*I*)

#### Refinement

*R*[*F* ^2^ > 2σ(*F* ^2^)] = 0.050
*wR*(*F* ^2^) = 0.144
*S* = 1.05
1655 reflections
111 parameters
H-atom parameters constrained
Δρ_max_ = 0.69 e Å^−3^
Δρ_min_ = −0.39 e Å^−3^

### 

Data collection: *SMART* (Bruker, 2001 ▶); cell refinement: *SAINT* (Bruker, 2001 ▶); data reduction: *SAINT*; program(s) used to solve structure: *SHELXTL* (Sheldrick, 2008 ▶); program(s) used to refine structure: *SHELXL97* (Sheldrick, 2008 ▶); molecular graphics: *ORTEPII* (Johnson, 1976 ▶); software used to prepare material for publication: *SHELXL97*.

## Supplementary Material

Crystal structure: contains datablocks global, I. DOI: 10.1107/S1600536808026627/lx2067sup1.cif

Structure factors: contains datablocks I. DOI: 10.1107/S1600536808026627/lx2067Isup2.hkl

Additional supplementary materials:  crystallographic information; 3D view; checkCIF report

## Figures and Tables

**Fig. 1.**
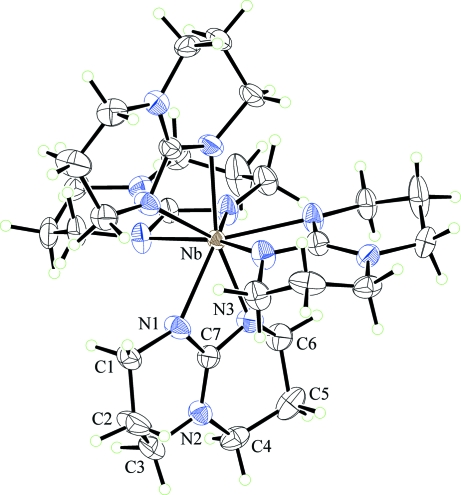
Molecular structure of the cation in (I) showing the crystallographic numbering scheme. Displacement ellipsoids are shown at the 35% probability level. The Nb atom is located on a site of symmetry 4.

## supplementary crystallographic information

### Comment

The title complex, [Nb(hpp)_4_][PF_6_] (I), feaures a [Nb(hpp)_4_]^+^ cation, with the Nb atom located on a site of symmetry 4, and a [PF_6_]^-^ anion, with fourfold symmetry; where hpp is 1,3,4,6,7,8-hexahydro-2*H*-pyrimido(1,2 - a)pyrimidine. The Nb atom is chelated four hpp ligands and the N_8_ donor set defines an approximate dodecahedral coordination environment (Fig. 1).

The conformations of the N1- and N-2 containing six-membered rings is twisted chair. Such a binding mode as observed in (I) is uncommon for the hpp ligand, which normally acts as a bridging group in various paddlewheel complexes (Cotton *et al.*, 2005). A related example of hpp acting as a chelating ligand is [Ta(hpp)_4_][Ta(CO)_6_] (Cotton *et al.*, 2000). Both complexes were obtained by oxidizing the precursors Nb_2_(hpp)_4_ and [Et_4_N][Ta(CO)_6_], respectively. The chelating mode of hpp is also found in some Ti complexes (Coles & Hitchcok, 2001).

### Experimental

The title complex (I) was obtained unintentionally in an attempt to oxidize the paddlewheel complex Nb_2_(hpp)_4_ with [Cp_2_Fe][PF_6_] in CH_2_Cl_2_. X-ray quality crystals were obtained by slow diffusion of hexanes into a CH_2_Cl_2_ solution of (I) at room temperature.

### Refinement

The H atoms were geometrically placed (C—H = 0.98 Å) and refined as riding with *U*_iso_(H) = 1.2*U*_eq_(C).

### Crystal data

**Table:**

[Nb(C_7_H_12_N_3_)_4_]PF_6_	*Z* = 2
*M**_r_* = 790.66	*F*_000_ = 820
Tetragonal, *P*4/*n*	*D*_x_ = 1.566 Mg m^−^^3^
Hall symbol: -P 4a	Mo *K*α radiation λ = 0.71069 Å
*a* = 13.531 (6) Å	Cell parameters from 10356 reflections
*b* = 13.531 (6) Å	θ = 2.1–27.5º
*c* = 9.159 (4) Å	µ = 0.48 mm^−^^1^
α = 90º	*T* = 213 (2) K
β = 90º	Block, yellow
γ = 90º	0.20 × 0.15 × 0.10 mm
*V* = 1676.9 (13) Å^3^	

### Data collection

**Table:**

Bruker SMART 1K CCD area-detector diffractometer	1655 independent reflections
Radiation source: fine-focus sealed tube	1381 reflections with *I* > 2σ(*I*)
Monochromator: graphite	*R*_int_ = 0.025
Detector resolution: 10 pixels mm^-1^	θ_max_ = 26.0º
*T* = 213(2) K	θ_min_ = 2.1º
ω and φ scans	*h* = −17→12
Absorption correction: multi-scan(SADABS; Sheldrick, 2004)	*k* = −17→16
*T*_min_ = 0.910, *T*_max_ = 0.953	*l* = −10→11
10356 measured reflections	

### Refinement

**Table:**

Refinement on *F*^2^	Secondary atom site location: difference Fourier map
Least-squares matrix: full	Hydrogen site location: inferred from neighbouring sites
*R*[*F*^2^ > 2σ(*F*^2^)] = 0.050	H-atom parameters constrained
*wR*(*F*^2^) = 0.144	*w* = 1/[σ^2^(*F*_o_^2^) + (0.067*P*)^2^ + 3.4248*P*] where *P* = (*F*_o_^2^ + 2*F*_c_^2^)/3
*S* = 1.05	(Δ/σ)_max_ < 0.001
1655 reflections	Δρ_max_ = 0.69 e Å^−^^3^
111 parameters	Δρ_min_ = −0.39 e Å^−^^3^
Primary atom site location: structure-invariant direct methods	Extinction correction: none

### Special details

**Table:**

Geometry. All esds (except the esd in the dihedral angle between two l.s. planes) are estimated using the full covariance matrix. The cell esds are taken into account individually in the estimation of esds in distances, angles and torsion angles; correlations between esds in cell parameters are only used when they are defined by crystal symmetry. An approximate (isotropic) treatment of cell esds is used for estimating esds involving l.s. planes.
Refinement. Refinement of F^2^ against ALL reflections. The weighted R-factor wR and goodness of fit S are based on F^2^, conventional R-factors R are based on F, with F set to zero for negative F^2^. The threshold expression of F^2^ > 2sigma(F^2^) is used only for calculating R-factors(gt) etc. and is not relevant to the choice of reflections for refinement. R-factors based on F^2^ are statistically about twice as large as those based on F, and R- factors based on ALL data will be even larger.

### Fractional atomic coordinates and isotropic or equivalent isotropic displacement parameters (Å^2^)

**Table:**

	*x*	*y*	*z*	*U*_iso_*/*U*_eq_	
Nb	0.7500	0.2500	0.5000	0.0388 (2)	
N1	0.6548 (3)	0.1964 (3)	0.3195 (4)	0.0670 (11)	
N2	0.6131 (3)	0.1782 (3)	0.5468 (4)	0.0677 (11)	
N3	0.4885 (3)	0.1508 (3)	0.3731 (4)	0.0598 (9)	
C1	0.6352 (4)	0.1994 (4)	0.1664 (5)	0.0730 (14)	
H1A	0.6931	0.1747	0.1137	0.088*	
H1B	0.6245	0.2682	0.1370	0.088*	
C2	0.5483 (5)	0.1405 (7)	0.1244 (6)	0.116 (3)	
H2A	0.5295	0.1587	0.0247	0.139*	
H2B	0.5675	0.0706	0.1231	0.139*	
C3	0.4618 (4)	0.1511 (4)	0.2179 (6)	0.0783 (15)	
H3A	0.4158	0.0967	0.1985	0.094*	
H3B	0.4281	0.2132	0.1945	0.094*	
C4	0.4136 (3)	0.1333 (4)	0.4827 (6)	0.0694 (14)	
H4A	0.3720	0.0777	0.4523	0.083*	
H4B	0.3713	0.1919	0.4909	0.083*	
C5	0.4580 (4)	0.1111 (4)	0.6279 (6)	0.0839 (18)	
H5A	0.4830	0.0431	0.6279	0.101*	
H5B	0.4067	0.1159	0.7032	0.101*	
C6	0.5406 (4)	0.1802 (4)	0.6648 (5)	0.0745 (14)	
H6A	0.5149	0.2474	0.6772	0.089*	
H6B	0.5718	0.1599	0.7566	0.089*	
C7	0.5805 (3)	0.1735 (3)	0.4103 (5)	0.0549 (10)	
P1	0.2500	0.2500	0.9214 (2)	0.0475 (5)	
F1	0.13862 (19)	0.2132 (2)	0.9219 (3)	0.0772 (9)	
F2	0.2500	0.2500	0.7468 (5)	0.0695 (14)	
F3	0.2500	0.2500	1.0963 (5)	0.0562 (11)	

### Atomic displacement parameters (Å^2^)

**Table:**

	*U*^11^	*U*^22^	*U*^33^	*U*^12^	*U*^13^	*U*^23^
Nb	0.0438 (3)	0.0438 (3)	0.0288 (4)	0.000	0.000	0.000
N1	0.056 (2)	0.107 (3)	0.0386 (18)	−0.013 (2)	−0.0009 (16)	−0.0025 (19)
N2	0.061 (2)	0.095 (3)	0.0472 (19)	−0.019 (2)	0.0110 (18)	−0.003 (2)
N3	0.051 (2)	0.067 (2)	0.062 (2)	0.0073 (17)	−0.0030 (17)	0.0021 (19)
C1	0.077 (3)	0.099 (4)	0.043 (2)	−0.014 (3)	−0.009 (2)	0.005 (2)
C2	0.084 (4)	0.217 (8)	0.047 (3)	−0.053 (5)	−0.014 (3)	0.001 (4)
C3	0.061 (3)	0.096 (4)	0.078 (3)	−0.001 (3)	−0.023 (3)	0.010 (3)
C4	0.048 (2)	0.055 (3)	0.105 (4)	−0.0021 (19)	0.012 (3)	−0.006 (3)
C5	0.090 (4)	0.072 (3)	0.089 (4)	−0.004 (3)	0.054 (3)	0.000 (3)
C6	0.076 (3)	0.101 (4)	0.047 (2)	−0.013 (3)	0.018 (2)	0.000 (3)
C7	0.055 (2)	0.065 (3)	0.045 (2)	−0.005 (2)	0.0064 (18)	0.0035 (19)
P1	0.0530 (7)	0.0530 (7)	0.0365 (10)	0.000	0.000	0.000
F1	0.0582 (15)	0.112 (2)	0.0619 (17)	−0.0192 (15)	0.0028 (13)	−0.0233 (16)
F2	0.087 (2)	0.087 (2)	0.035 (2)	0.000	0.000	0.000
F3	0.0660 (18)	0.0660 (18)	0.036 (2)	0.000	0.000	0.000

### Geometric parameters (Å, °)

**Table:**

Nb—N2	2.135 (4)	C2—H2B	0.9800
Nb—N2^i^	2.135 (4)	C3—H3A	0.9800
Nb—N1	2.218 (4)	C3—H3B	0.9800
Nb—N1^i^	2.218 (4)	C4—C5	1.490 (8)
Nb—C7	2.648 (4)	C4—H4A	0.9800
Nb—C7^i^	2.648 (4)	C4—H4B	0.9800
N1—C7	1.341 (5)	C5—C6	1.496 (8)
N1—C1	1.428 (5)	C5—H5A	0.9800
N2—C7	1.328 (6)	C5—H5B	0.9800
N2—C6	1.460 (6)	C6—H6A	0.9800
N3—C7	1.326 (5)	C6—H6B	0.9800
N3—C4	1.447 (6)	P1—F1	1.587 (3)
N3—C3	1.466 (6)	P1—F1^ii^	1.587 (3)
C1—C2	1.472 (7)	P1—F1^iii^	1.587 (3)
C1—H1A	0.9800	P1—F1^iv^	1.587 (3)
C1—H1B	0.9800	P1—F2	1.600 (5)
C2—C3	1.457 (8)	P1—F3	1.602 (5)
C2—H2A	0.9800		
			
N2—Nb—N2^i^	156.8 (2)	C7—N1—C1	118.4 (4)
N2—Nb—N2^v^	92.31 (4)	C7—N1—Nb	92.8 (3)
N2^i^—Nb—N2^v^	92.31 (4)	C1—N1—Nb	146.2 (3)
N2—Nb—N2^vi^	92.31 (4)	C7—N2—C6	118.3 (4)
N2^i^—Nb—N2^vi^	92.31 (4)	C7—N2—Nb	97.0 (3)
N2^v^—Nb—N2^vi^	156.8 (2)	C6—N2—Nb	136.4 (3)
N2—Nb—N1	59.80 (14)	C7—N3—C4	121.1 (4)
N2^i^—Nb—N1	143.38 (14)	C7—N3—C3	118.7 (4)
N2^v^—Nb—N1	80.23 (16)	C4—N3—C3	120.0 (4)
N2^vi^—Nb—N1	82.54 (17)	N1—C1—C2	112.9 (4)
N2—Nb—N1^v^	82.54 (17)	N1—C1—H1A	109.0
N2^i^—Nb—N1^v^	80.23 (16)	C2—C1—H1A	109.0
N2^v^—Nb—N1^v^	59.80 (14)	N1—C1—H1B	109.0
N2^vi^—Nb—N1^v^	143.38 (14)	C2—C1—H1B	109.0
N1—Nb—N1^v^	123.75 (12)	H1A—C1—H1B	107.8
N2—Nb—N1^vi^	80.23 (16)	C3—C2—C1	115.7 (6)
N2^i^—Nb—N1^vi^	82.54 (17)	C3—C2—H2A	108.3
N2^v^—Nb—N1^vi^	143.38 (14)	C1—C2—H2A	108.3
N2^vi^—Nb—N1^vi^	59.80 (14)	C3—C2—H2B	108.3
N1—Nb—N1^vi^	123.75 (12)	C1—C2—H2B	108.3
N1^v^—Nb—N1^vi^	83.6 (2)	H2A—C2—H2B	107.4
N2—Nb—N1^i^	143.38 (14)	C2—C3—N3	111.8 (4)
N2^i^—Nb—N1^i^	59.80 (14)	C2—C3—H3A	109.3
N2^v^—Nb—N1^i^	82.54 (17)	N3—C3—H3A	109.3
N2^vi^—Nb—N1^i^	80.23 (16)	C2—C3—H3B	109.3
N1—Nb—N1^i^	83.6 (2)	N3—C3—H3B	109.3
N1^v^—Nb—N1^i^	123.75 (12)	H3A—C3—H3B	107.9
N1^vi^—Nb—N1^i^	123.75 (12)	N3—C4—C5	111.7 (4)
N2—Nb—C7	29.86 (14)	N3—C4—H4A	109.3
N2^i^—Nb—C7	172.73 (14)	C5—C4—H4A	109.3
N2^v^—Nb—C7	89.57 (16)	N3—C4—H4B	109.3
N2^vi^—Nb—C7	83.26 (16)	C5—C4—H4B	109.3
N1—Nb—C7	30.39 (13)	H4A—C4—H4B	107.9
N1^v^—Nb—C7	106.74 (15)	C4—C5—C6	112.1 (4)
N1^vi^—Nb—C7	100.07 (15)	C4—C5—H5A	109.2
N1^i^—Nb—C7	113.57 (13)	C6—C5—H5A	109.2
N2—Nb—C7^vi^	89.57 (15)	C4—C5—H5B	109.2
N2^i^—Nb—C7^vi^	83.26 (16)	C6—C5—H5B	109.2
N2^v^—Nb—C7^vi^	172.73 (14)	H5A—C5—H5B	107.9
N2^vi^—Nb—C7^vi^	29.86 (14)	N2—C6—C5	108.9 (4)
N1—Nb—C7^vi^	106.74 (15)	N2—C6—H6A	109.9
N1^v^—Nb—C7^vi^	113.57 (13)	C5—C6—H6A	109.9
N1^vi^—Nb—C7^vi^	30.39 (13)	N2—C6—H6B	109.9
N1^i^—Nb—C7^vi^	100.07 (15)	C5—C6—H6B	109.9
C7—Nb—C7^vi^	95.53 (5)	H6A—C6—H6B	108.3
N2—Nb—C7^v^	83.26 (16)	N3—C7—N2	124.4 (4)
N2^i^—Nb—C7^v^	89.57 (15)	N3—C7—N1	126.7 (4)
N2^v^—Nb—C7^v^	29.86 (14)	N2—C7—N1	108.8 (4)
N2^vi^—Nb—C7^v^	172.73 (14)	N3—C7—Nb	169.6 (3)
N1—Nb—C7^v^	100.07 (15)	N2—C7—Nb	53.2 (2)
N1^v^—Nb—C7^v^	30.39 (13)	N1—C7—Nb	56.8 (2)
N1^vi^—Nb—C7^v^	113.57 (13)	F1—P1—F1^ii^	90.000 (2)
N1^i^—Nb—C7^v^	106.74 (15)	F1—P1—F1^iii^	179.7 (2)
C7—Nb—C7^v^	95.53 (5)	F1^ii^—P1—F1^iii^	90.000 (2)
C7^vi^—Nb—C7^v^	143.83 (18)	F1—P1—F1^iv^	90.000 (1)
N2—Nb—C7^i^	172.73 (14)	F1^ii^—P1—F1^iv^	179.7 (2)
N2^i^—Nb—C7^i^	29.86 (14)	F1^iii^—P1—F1^iv^	90.000 (2)
N2^v^—Nb—C7^i^	83.26 (16)	F1—P1—F2	90.16 (12)
N2^vi^—Nb—C7^i^	89.57 (16)	F1^ii^—P1—F2	90.16 (12)
N1—Nb—C7^i^	113.57 (13)	F1^iii^—P1—F2	90.16 (12)
N1^v^—Nb—C7^i^	100.07 (15)	F1^iv^—P1—F2	90.16 (12)
N1^vi^—Nb—C7^i^	106.74 (15)	F1—P1—F3	89.84 (12)
N1^i^—Nb—C7^i^	30.39 (13)	F1^ii^—P1—F3	89.84 (12)
C7—Nb—C7^i^	143.83 (18)	F1^iii^—P1—F3	89.84 (12)
C7^vi^—Nb—C7^i^	95.53 (5)	F1^iv^—P1—F3	89.84 (12)
C7^v^—Nb—C7^i^	95.53 (5)	F2—P1—F3	180.000 (2)
			
N2—Nb—N1—C7	7.7 (3)	N3—C4—C5—C6	45.0 (6)
N2^i^—Nb—N1—C7	−173.2 (3)	C7—N2—C6—C5	39.2 (7)
N2^v^—Nb—N1—C7	106.2 (3)	Nb—N2—C6—C5	179.1 (4)
N2^vi^—Nb—N1—C7	−89.4 (3)	C4—C5—C6—N2	−54.6 (6)
N1^v^—Nb—N1—C7	63.0 (4)	C4—N3—C7—N2	3.0 (7)
N1^vi^—Nb—N1—C7	−43.6 (4)	C3—N3—C7—N2	178.2 (5)
N1^i^—Nb—N1—C7	−170.3 (4)	C4—N3—C7—N1	−176.2 (5)
C7^vi^—Nb—N1—C7	−71.7 (3)	C3—N3—C7—N1	−1.0 (7)
C7^v^—Nb—N1—C7	83.7 (2)	C4—N3—C7—Nb	−70.1 (19)
C7^i^—Nb—N1—C7	−175.65 (18)	C3—N3—C7—Nb	105.1 (17)
N2—Nb—N1—C1	165.9 (7)	C6—N2—C7—N3	−14.1 (7)
N2^i^—Nb—N1—C1	−15.0 (8)	Nb—N2—C7—N3	−167.5 (4)
N2^v^—Nb—N1—C1	−95.6 (7)	C6—N2—C7—N1	165.2 (5)
N2^vi^—Nb—N1—C1	68.8 (7)	Nb—N2—C7—N1	11.8 (4)
N1^v^—Nb—N1—C1	−138.8 (6)	C6—N2—C7—Nb	153.4 (5)
N1^vi^—Nb—N1—C1	114.6 (6)	C1—N1—C7—N3	1.6 (8)
N1^i^—Nb—N1—C1	−12.1 (6)	Nb—N1—C7—N3	168.0 (4)
C7—Nb—N1—C1	158.2 (9)	C1—N1—C7—N2	−177.7 (5)
C7^vi^—Nb—N1—C1	86.5 (7)	Nb—N1—C7—N2	−11.3 (4)
C7^v^—Nb—N1—C1	−118.0 (7)	C1—N1—C7—Nb	−166.4 (5)
C7^i^—Nb—N1—C1	−17.4 (7)	N2—Nb—C7—N3	80.4 (17)
N2^i^—Nb—N2—C7	173.5 (3)	N2^v^—Nb—C7—N3	175.8 (17)
N2^v^—Nb—N2—C7	−85.1 (3)	N2^vi^—Nb—C7—N3	−26.3 (17)
N2^vi^—Nb—N2—C7	72.2 (3)	N1—Nb—C7—N3	−113.0 (18)
N1—Nb—N2—C7	−7.8 (3)	N1^v^—Nb—C7—N3	117.6 (17)
N1^v^—Nb—N2—C7	−144.2 (3)	N1^vi^—Nb—C7—N3	31.3 (18)
N1^vi^—Nb—N2—C7	131.0 (3)	N1^i^—Nb—C7—N3	−102.5 (17)
N1^i^—Nb—N2—C7	−4.5 (5)	C7^vi^—Nb—C7—N3	1.0 (17)
C7^vi^—Nb—N2—C7	101.9 (3)	C7^v^—Nb—C7—N3	146.5 (18)
C7^v^—Nb—N2—C7	−113.6 (3)	C7^i^—Nb—C7—N3	−106.3 (18)
N2^i^—Nb—N2—C6	28.4 (5)	N2^v^—Nb—C7—N2	95.4 (3)
N2^v^—Nb—N2—C6	129.7 (6)	N2^vi^—Nb—C7—N2	−106.7 (3)
N2^vi^—Nb—N2—C6	−73.0 (5)	N1—Nb—C7—N2	166.6 (5)
N1—Nb—N2—C6	−153.0 (6)	N1^v^—Nb—C7—N2	37.2 (3)
N1^v^—Nb—N2—C6	70.6 (5)	N1^vi^—Nb—C7—N2	−49.1 (3)
N1^vi^—Nb—N2—C6	−14.2 (5)	N1^i^—Nb—C7—N2	177.1 (3)
N1^i^—Nb—N2—C6	−149.7 (5)	C7^v^—Nb—C7—N2	66.1 (3)
C7—Nb—N2—C6	−145.2 (7)	C7^i^—Nb—C7—N2	173.3 (3)
C7^vi^—Nb—N2—C6	−43.3 (5)	N2—Nb—C7—N1	−166.6 (5)
C7^v^—Nb—N2—C6	101.2 (5)	N2^v^—Nb—C7—N1	−71.2 (3)
C7—N1—C1—C2	−22.7 (8)	N2^vi^—Nb—C7—N1	86.7 (3)
Nb—N1—C1—C2	−177.8 (5)	N1^v^—Nb—C7—N1	−129.3 (3)
N1—C1—C2—C3	44.3 (9)	N1^vi^—Nb—C7—N1	144.4 (3)
C1—C2—C3—N3	−43.2 (8)	N1^i^—Nb—C7—N1	10.5 (4)
C7—N3—C3—C2	21.6 (8)	C7^vi^—Nb—C7—N1	114.0 (3)
C4—N3—C3—C2	−163.1 (5)	C7^v^—Nb—C7—N1	−100.5 (3)
C7—N3—C4—C5	−19.0 (6)	C7^i^—Nb—C7—N1	6.8 (3)
C3—N3—C4—C5	165.8 (5)		

Symmetry codes: (i) −*x*+3/2, −*y*+1/2, *z*; (ii) −*y*+1/2, *x*, *z*; (iii) −*x*+1/2, −*y*+1/2, *z*; (iv) *y*, −*x*+1/2, *z*; (v) −*y*+1, *x*−1/2, −*z*+1; (vi) *y*+1/2, −*x*+1, −*z*+1.
